# Copy number alterations in small intestinal neuroendocrine tumors determined by array comparative genomic hybridization

**DOI:** 10.1186/1471-2407-13-505

**Published:** 2013-10-29

**Authors:** Jamileh Hashemi, Omid Fotouhi, Luqman Sulaiman, Magnus Kjellman, Anders Höög, Jan Zedenius, Catharina Larsson

**Affiliations:** 1 Department of Oncology-Pathology, Karolinska Institutet, Cancer Center Karolinska, Karolinska University Hospital R8:04, Stockholm SE-171 76, Sweden; 2 Department of Molecular Medicine and Surgery, Endocrine Surgery Unit, Karolinska Institutet, Karolinska University Hospital P9:03, Stockholm SE-171 76, Sweden; 3 Department of Breast and Endocrine Surgery, Karolinska University Hospital, Stockholm SE-171 76, Sweden

**Keywords:** Small intestine, Neuroendocrine tumor, Carcinoid, Array CGH, Chromosome 18

## Abstract

**Background:**

Small intestinal neuroendocrine tumors (SI-NETs) are typically slow-growing tumors that have metastasized already at the time of diagnosis. The purpose of the present study was to further refine and define regions of recurrent copy number (CN) alterations (CNA) in SI-NETs.

**Methods:**

Genome-wide CNAs was determined by applying array CGH (a-CGH) on SI-NETs including 18 primary tumors and 12 metastases. Quantitative PCR analysis (qPCR) was used to confirm CNAs detected by a-CGH as well as to detect CNAs in an extended panel of SI-NETs. Unsupervised hierarchical clustering was used to detect tumor groups with similar patterns of chromosomal alterations based on recurrent regions of CN loss or gain. The log rank test was used to calculate overall survival. Mann–Whitney *U* test or Fisher’s exact test were used to evaluate associations between tumor groups and recurrent CNAs or clinical parameters.

**Results:**

The most frequent abnormality was loss of chromosome 18 observed in 70% of the cases. CN losses were also frequently found of chromosomes 11 (23%), 16 (20%), and 9 (20%), with regions of recurrent CN loss identified in 11q23.1-qter, 16q12.2-qter, 9pter-p13.2 and 9p13.1-11.2. Gains were most frequently detected in chromosomes 14 (43%), 20 (37%), 4 (27%), and 5 (23%) with recurrent regions of CN gain located to 14q11.2, 14q32.2-32.31, 20pter-p11.21, 20q11.1-11.21, 20q12-qter, 4 and 5. qPCR analysis confirmed most CNAs detected by a-CGH as well as revealed CNAs in an extended panel of SI-NETs. Unsupervised hierarchical clustering of recurrent regions of CNAs revealed two separate tumor groups and 5 chromosomal clusters. Loss of chromosomes 18, 16 and 11 and again of chromosome 20 were found in both tumor groups. Tumor group II was enriched for alterations in chromosome cluster-d, including gain of chromosomes 4, 5, 7, 14 and gain of 20 in chromosome cluster-b. Gain in 20pter-p11.21 was associated with short survival. Statistically significant differences were observed between primary tumors and metastases for loss of 16q and gain of 7.

**Conclusion:**

Our results revealed recurrent CNAs in several candidate regions with a potential role in SI-NET development. Distinct genetic alterations and pathways are involved in tumorigenesis of SI-NETs.

## Background

Small intestinal neuroendocrine tumor (SI-NET) arising from enterochromaffin cells is the most common type of gastrointestinal endocrine tumor. SI-NET is also termed midgut carcinoids, well-differentiated neuroendocrine tumor of the midgut, ileal carcinoid or neuroendocrine tumor of the midgut [1,2]. The tumors are usually slow-growing in nature [3,4]. SI-NETs are mostly sporadic, however, a few small families with a history of the disease have recently been characterized [5,6]. Many patients are asymptomatic and the disease can be indolent for many years and diagnosed incidentally. Approximately 20% of patients present with carcinoid syndrome, a clinical entity characterized by flushing, diarrhea, abdominal pain, cardiac valvular fibrosis and bronchial constriction [7,8]. The clinical picture of the carcinoid syndrome, which usually occur in patients with liver metastases, is due to excessive production and release of hormones and substances such as serotonin, tachykinins and prostaglandins [9]. Metastases are first recognized in regional lymph nodes followed by the liver and less frequently in the ovaries or other distant sites [3,10]. Surgery is the first treatment of choice, however, in patients with metastatic disease surgical treatment seldom leads to cure [11].

Several molecular cytogenetic studies have been performed with the aim of understanding the mechanisms of SI-NET development. Using conventional comparative genomic hybridization (CGH) we and others observed frequent copy number (CN) losses at 18q, 11q, 16q, and gains of 4p [12,13]. The most common aberration, loss of 18, was also identified by loss of heterozygosity (LOH) screening [14] and has been validated in SI-NET by several other groups using array CGH (a-CGH) or single nucleotide polymorphism based arrays (SNP-arrays) [5,15-17]. These findings suggest the location of putative tumor suppressor gene(s) for SI-NET development in chromosome 18. In addition, the possibility of two or more genetically distinct groups in SI-NET has been suggested, namely those characterized by loss of chromosome 18 and a second group with gain of chromosomes 4, 5, 7 and 14 [15,16]. SI-NETs with gain of chromosome 14 were reported to have intact chromosome 18 and poor prognosis [15]. However, so far no target gene or genes have been identified for the frequent genetic aberrations in SI-NETs.

To further refine and define regions of recurrent CN aberrations (CNAs) we applied a-CGH and genomic quantitative real-time PCR (qPCR) to a panel of SI-NETs from 32 patients and evaluated the findings to the clinical parameters and patient outcome. We also aimed to identify differences in CNA profiles between primary tumors and metastases.

## Methods

### Tumor material

Fresh frozen samples from patients with sporadic SI-NETs were included in this study. All tumor samples were obtained from patients operated for SI-NETs at the Karolinska University Hospital-Solna since 1989 and had been collected and stored at the Karolinska tissue biobank. All samples were obtained with informed oral consent and ethical approval from the local ethical committee of Karolinska Institutet. A standardized procedure of tumor collection was employed including macroscopical dissection by a histopathologist, snap freezing and storage at −80°C until use. The histopathological diagnosis was established at routine examination according to published criteria [9,18]. The immunohistochemical examination included staining for chromogranin A [19], and/or Grimelius silver staining and Masson staining as a marker for serotonin [12].

Totally 30 tumor samples (19 primary tumors and 11 metastases) from 29 patients (15 females and 14 males) were collected for the a-CGH screening. For the subsequent qPCR analyses of selected loci the tumor sample panel was extended to a total of 43 tumor samples from 32 patients. The clinical data concerning gender, age at diagnosis of the primary tumor, functioning tumor, previous SI-NET surgery, metastasis and follow-up are detailed in Additional file 1: Table S1. Eighteen tumors from 18 patients were from our previous publication (cases 1–18, Table 1) [12]. Twenty-four of the samples were primary tumors and 19 were metastases. Twelve of the metastases were regional (mesenterial, regional lymph nodes or omental) and 7 were distant metastases (5 liver and two ovarian). Matched primary and metastatic lesions were available from 9 out of 32 cases, and for two cases two metastatic samples were included. A sample of non-neoplastic tissue close to the primary tumor from a SI-NET case was included as normal control for quantitative real time PCR (qTR-PCR). Sixteen cases were female and 16 were male. The median age at the time of surgery was 67.5 years (mean 65 years; range 39–80 years). Patients were followed-up from diagnosis until death or last date of contact for a median of 88 months (mean 94 months; range 7–222). Seventeen patients were known to have a functioning tumor based on carcinoid syndrome and/or increased levels of 5-HIAA. Seven patients had been treated surgically before the operation at which the sample studied was collected. In addition, cases 1–8 were operated before the introduction of somatostatin analogues, which was subsequently the standard peroperative treatment concerning cases 9–32. MIB-1 proliferation index was available for cases 19–32 which all showed <2% except in case 25 that showed 2.5%. In cases 1–18 the primary tumor was operated in the period 1986–1997 i.e. before the introduction of MIB-1 analysis in the clinical workup of these tumors.

**Table 1:** Recurrent copy number losses detected by a-CGH in SI-NETs

**Case no.**	**P /**					
	**/ M**	**Chr 9**	**Chr 11**	**Chr 13**	**Chr 16**	**Chr 18**
1	M	-	11q14.1-ter	-	16q12.1-ter	-
3	P	9	-	13q11-ter	-	18
4	p	-	-	-	-	18
5	M	-	-	-	-	18
6	M	-	-	-	16q12.2-ter	18
7	P	-	11q14.1-ter	13q33.1	-	18p11.31; q12.1-ter
8	P	-	-	-	-	-
9	M	-	11q21-ter	-	16q12.1-ter	18
10	P		-	-	-	18
11	P	9pter-13.2	11q13.4; q14.1-ter	-	-	18pter-q21.31
12	P	-	-	-	-	18
13	P	-	(11q23.1q-ter)	-	-	(18)pter-11.23
14	P	-		-	-	-
15	P	-	-	-	-	18
16	P	-	-	-	-	18q22.1
17	P	-	-	-	-	(18pter-11.21; q11.2-23)
18	P	(9p13.1-q13.2)	-	13q14.11-21.32	-	-
20	P	-	-	-	-	-
21	M	-	(11q13.3-ter) q13.3-13.5; q23.3-ter	-	16p11.2; p11.2-11.1; q12.1-ter	(18)pter-q11.2; q12.3-ter
23	M	-	-	-	-	-
24	M	9	-	13	-	18
25	P	-	-	-	-	-
26	P	-	-	-	16p11.2	18
27	P	-	11q22.1-ter	-	16q12.1-ter	18pter-11.21
27	M	-	-	-	-	-
28	M	-	-	-	-	(18)18pter-11.23; q11.1-22.4
29	P	-	-	-	-	-
30	M	-	-	-	-	18
31	P	(9p13.1-q12)	-	-	-	18p11.31
32	M	9p13.1-11.2	-	-	-	18

P = primary tumor; M = metastasis; chr= chromosome; Alterations written within parenthesis represent borderline alterations at the threshold.

### DNA extraction

Genomic DNA was either available from our published series of SI-NETs [12] or was isolated from frozen tissue using DNeasy Blood and Tissue DNA isolation kit (Qiagen GmbH, Hilden, Germany) according to the manufacturer’s protocol. The DNA quality and concentration was assessed using a NanoDrop A100 Spectrophotometer (ND-1000, Thermo Scientific, USA).

### Array CGH (a-CGH) and data analysis

A-CGH was applied on 30 SI-NETs from 29 patients using human BAC (Bacterial Artificial Chromosome) arrays with 1 Mb resolution for 6 samples (cases 1, 3, 4, 6, 7, and 9), or of tiling type for 27 samples from 26 cases (1, 2, 4, 5, 6, 8, 10–32). Initially, we used commercially available 1 Mb arrays (Spectral Genomics, Houston, TX USA currently Perkin Elmer) applying previously described methodology [20]. Briefly, a dye swap method was applied, and the Spectralware 2 software (Spectral Genomics) was used for data analysis with cut-off levels at 1.2 and 0.8 for identification of gain or loss, respectively. Subsequently, human tiling 33 K and 38 K BAC arrays, produced at the SCIBLU Genomics Centre at Lund University, Sweden (http://www.lu.se/sciblu) were used. The 33 K and 38 K array slides contained 33,370 and 38,000 BAC clones, respectively (CHORI BACPAC resources) (http://bacpac.chori.org/ genomicRearrays.php) giving a resolution of one clone per 50–100 kb. Information about experimental procedures and data analyses have previously been described in detail [21]. After hybridization, slides were scanned using a Genepix 4200A scanner (Axon instruments Inc., Union City, CA). The resulting Tiff images were quantified using the GenePix Pro 6.0 package analysis software (Axon instruments, Wheatherford TX, USA) and gene pix result (GPR) files were generated. The GPR files were then loaded onto BioArray Software Environment (BASE; http://base.thep.lu.se/) [22] for further analyses and data processing such as filtering, normalization, smoothing and profiling.

Log_2_ ratio thresholds were used to define gain (+0.25), loss (−0.25), amplification (+1) and homozygous loss (−1). Software based profiles were inspected manually for confirmation of CNAs. Recurrent regions of CNAs were defined as those observed in three or more a-CGH profiles [23]. Inside recurrent regions, minimal overlapping regions (MOR) were identified as the smallest region of overlapping loss or gain. CNA regions in telomeric and centromeric regions as well as involving few clones only were interpreted with caution. CNAs overlapping with known normal genomic variants according to the Database of Genomic Variants (http://dgvbeta.tcag.ca/dgv/app/home?ref=NCBI36/hg18) were not reported.

For cluster analysis of recurrent regions of CNAs, a binary matrix was generated where the rows indicated altered chromosomal regions and columns represented the tumor samples. In each case copy number alterations for each case CN status was coded as “0” for normal copy number or “1” for CNA either gain or loss. The matrix was uploaded in Multi Experiment Viewer 4.7.4 software and unsupervised hierarchical clustering analysis was performed using Euclidean distance and average linkage [24]. CNA data for case 27 with paired primary-metastasis samples were pooled.

### Genomic quantitative real-time PCR (qPCR)

qPCR analysis of CNs was applied to all 43 SI-NETs for selected loci with frequent CN losses in 18p (*EMILIN2*), 18q (*DCC, BCL2, CDH19*), 16q (*CDH1*) and 11q (*SDHD*). All target and reference assays were purchased from Applied Biosystems. *RNaseP* (on chromosome 14) was used as endogenous control for normalization of analyzed loci in chromosomes 18, 16 and 11. The following assays were used: *EMILIN2* (Hs01996822), *DCC* (Hs02317964, Hs02967342)*, BCL2* (Hs01500302)*, CDH19* (Hs02826809, Hs02956257), *CDH1* Hs00934267), *SDHD* (Hs03794135) and *RNaseP* (part number 4403326). Assays were chosen to avoid overlap with known SNPs. The experimental procedure recommended by the manufacturer (Applied Biosystems) was followed. Five nanogram genomic DNA was used in the qPCR reaction, and water was analyzed in parallel as negative control. All qPCR reactions were run in quadruplicate in a Step One Plus qRT-PCR machine (Applied Biosystems) using standard cycling conditions of 10 min at 95°C, followed by 40 cycles of [95°C for 15 sec and at 60°C for 1 min]. Pooled normal blood DNA (Promega, Madison, WI, USA) was used as calibrator and a normal mucosal intestine DNA as normal control. CNs were predicted by Copy Caller v1.0 software (Applied Biosystems).

### Statistical analysis

The follow-up period was calculated from the date of diagnosis of the primary tumor until the date of death or the last date of contact. The log rank test was used to calculate overall survival and illustrated by Kaplan-Meier plots concerning tumor groups, recurrent region of CNAs and clinical parameters. Moreover to evaluate the possible effect of confounding factors (e.g. gender or regional, distant and extra-hepatic metastases) multivariate analysis using Cox proportional hazards modeling was applied to those recurrent regions of CNAs which were significantly associated to survival. Associations between tumor groups and recurrent CNA or clinical parameters were evaluated by Fisher’s exact test and for age at diagnosis by Mann–Whitney *U* test. All statistical analyses were performed using the statistical Software SPSS v 16.0. *P*-values ≤ 0.05 were regarded as significant.

## Results

### Overall findings of copy number alterations detected by a-CGH

We determined genome-wide CNAs in 30 tumors representing 19 primary tumors and 11 metastases from 29 patients with SI-NET using a-CGH. All samples analyzed displayed CNAs, with the largest and smallest extent of total CNAs observed in tumors number 4 (550 Mb) and 29 (2.5 Mb), respectively. CN losses and gains were in many cases extensive and involved entire or almost entire chromosomes. Gains were more common than losses. Recurrent CN losses were found on chromosomes 18, 16, 11, 9 and 13 (Table 1) and gains on chromosomes 20, 14, 4, 5, and 7 (Table 2). Overall the CNAs detected by a-CGH in this study were consistent with those detected for the 17 cases analyzed by metaphase CGH in our previous study [12] as well as with those samples analyzed by the 1 Mb resolution array. However, a-CGH identified additional CNAs not previously detected by metaphase CGH such as loss of 18p in case 4, 10 and 15 or gains of chromosomes 4 and 5 in case 14.

**Table 2:** Recurrent copy number gains detected by a-CGH in SI-NETs

**Case no.**	**P /**					
	**/ M**	**Chr 4**	**Chr 5**	**Chr 7**	**Chr 14**	**Chr 20**
1	M	(4)pter-15.32	(5)pter-15.33; q23.3-ter	-	(14q11.1-21.3); q32.12-ter	20
3	P	-	-	-	-	-
4	P	(4)pter-15.2; p14-qter	(5)pter-15.1; p13.3-qter	-	-	(20)pter-q13.33
5	M	-	-	-	-	-
6	M	-	-	-	-	-
7	P	4	5	-	14	-
8	P	-	-	-	-	-
9	M	(4)	-	-	-	20q12-ter
10	P	-	-	-	-	-
11	P	-	-	-	-	-
12	P	-	-	-	-	-
13	P	-	-	-	-	-
14	P	(4)pter-16.1; p14-q12	(5)pter-14.3; q35.1-ter	-	-	(20)pter-13; q11.21-ter
15	P	-	-	-	-	20q13.32; qter
16	P	-	-	-	-	20q11.1-11.21; q13.33
17	P	-	-	-	-	-
18	P	-	-	-	-	-
20	P	-	-	-	14q11.2	-
21	M	-	-	7q22.3-ter	-	-
23	M	-	-	-	(14q11.2)	-
24	M	-	-	-	-	20
25	P	(4)p16.3-12; q22.1-ter	(5)pter-q35.2	-	(14) q11.1-32.2	-
26	P	-	-	-	-	(20)pter-11.21; q11.23-13.33
27	P	-	-	(7)pter-22.3; p15.3-q35; q35-ter	(14) q11.2; q23.3-ter	(20)pter-q13.2; q13.31-ter
27	M	-	-	-	(14q11.2)	-
28	M	4pter-16.1	5pter; q35.2-35.3	7p22.3; p22.2-22.1; q22.1; qter	14q11.2; q32.2-ter	20p12.1; q13.31-13.32; q13.33-ter
29	P	**-**	-	-	-	-
30	M	**-**	-	-	-	-
31	P	**-**	-	-	-	-
32	M	(4)q26-35.2	(5)pter-15.1; q11.1-13.1; q13.2-ter	(7)pter-22.2; p22.1-11.2; q11.23-ter	(14q11.1-32.31) q11.1	20pter-11.21; q11.23-ter

P = primary tumor; M = metastasis; chr= chromosome; Alterations written within parenthesis represent borderline alterations at the threshold.

### Copy number losses

**Figure 1:**
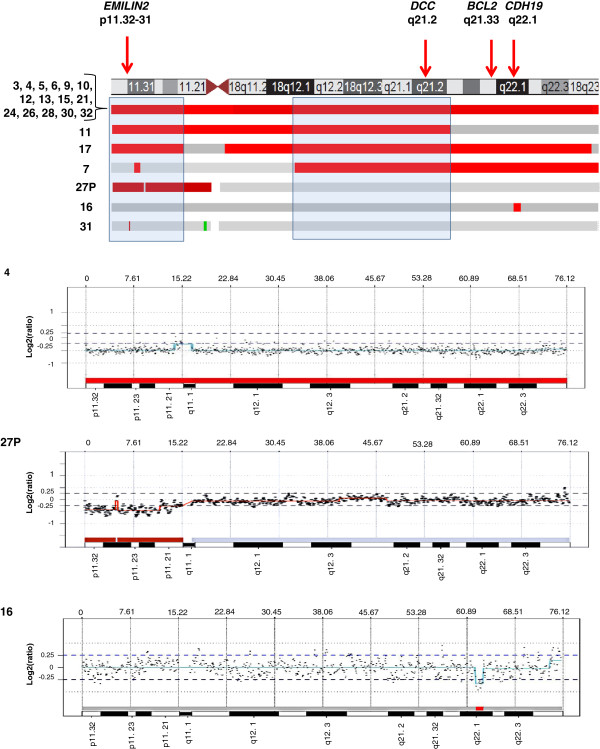
**Mapping of CN losses detected in chromosome 18 by a-CGH analysis.** At the top, the location of the *EMILIN2*, *DCC*, *BCL2*, *CDH19* genes analyzed by qPCR are indicated by arrows next to an ideogram of chromosome 18 (UCSC Genome Browser). Alterations in cases with detected CN losses are schematically illustrated by bars with red indicating losses and green marking gains. Fifteen samples exhibited loss of the entire chromosome 18, while samples 7, 11, 16, 17, 27P and 31 showed losses restricted to parts of the chromosome. Below are shown a-CGH profiles for case 4 with entire chromosome 18 loss, case 27P with deletion of 18pter-11.21, and case 16 with a 2 Mb deletion at 18q22.1.

Recurrent CN losses were most frequently found on chromosomes 18 (70%), 11q (23%), 9 (20%), 16 (17%) and 13 (13%). Fifteen of 30 tumors (50%) had entire or almost entire loss of chromosome 18. In addition, loss of chromosome 18 was the only detectable recurrent CNA in four tumors (5, 10, 12, and 17), three of which were primary tumors. Sub-chromosomal losses of chromosome 18 were observed in six tumors (Table 1, Figure 1). Two recurrent regions of loss were identified, one on 18p (pter-p11.21) and another on 18q (q12.1-q21.31) (Figure 1). A MOR of loss of 247 kb was observed within the 18pter-p11.21 interval at 18p11.32-p11.31 in tumor 31. This MOR encompasses three known genes, *KIAA0650*, *LPIN2* and *EMILIN2,* and the loss of the latter was verified by genomic qPCR. Another MOR of 2 Mb loss was identified in tumor 16 at 18q22.1 which encompasses the genes *CDH7* and *CDH19*. However, we could not confirm the loss of *CDH19* on 18q22.1 in tumor 16 by qPCR.

**Figure 2:**
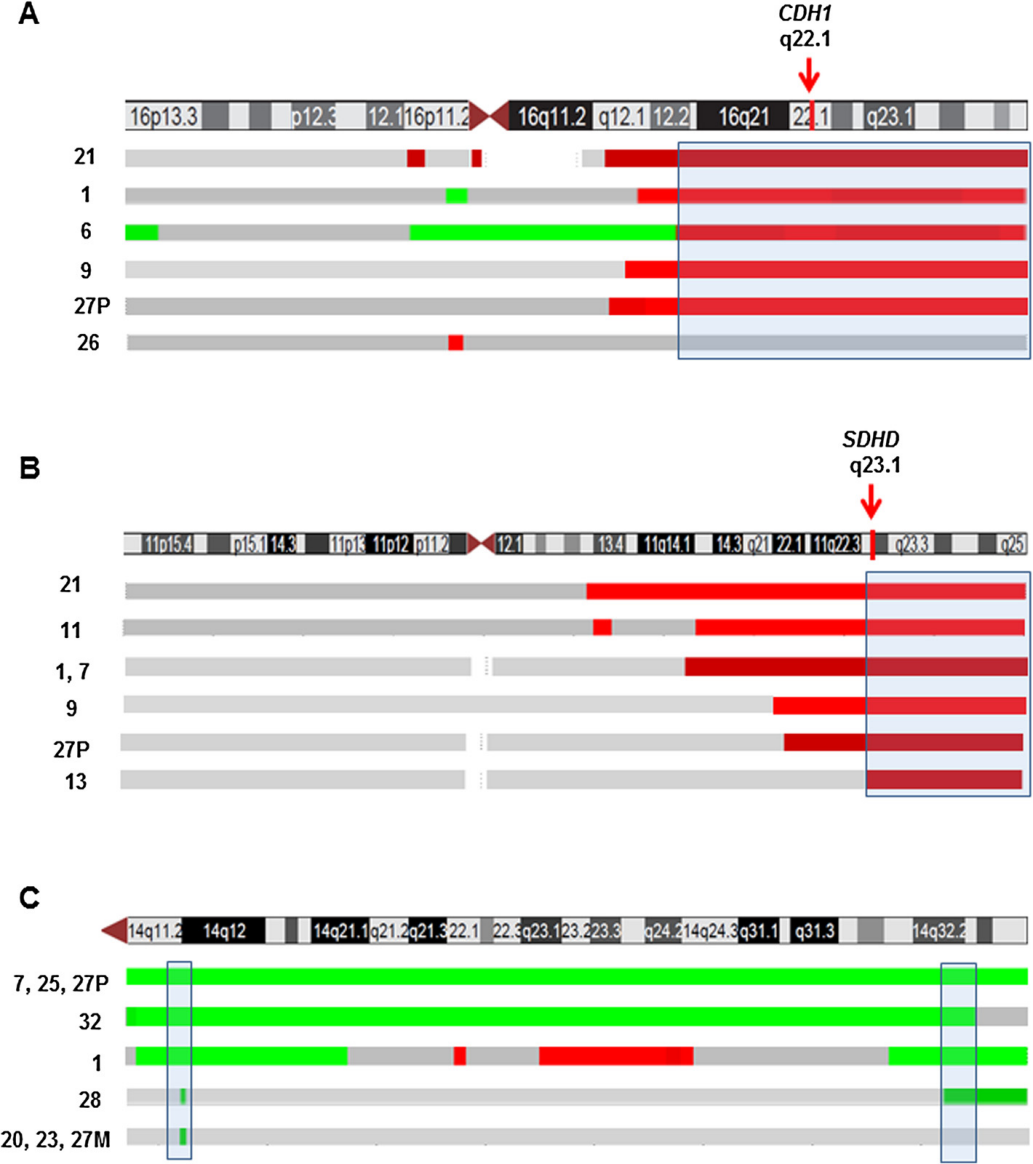
**Mapping of CN losses in (A) chromosomes 16 and (B) 11 (B) and (C) localization of CN gains in chromosome 14 by a-CGH.** For each chromosome is shown an ideogram (UCSC Genome Browser) together with bars indicating losses in green and gains in green. The genomic location of the *CDH1* and *SDHD* genes analyzed by qPCR are marked by arrows.

Recurrent CN losses were observed on chromosome 16 in 5/30 (17%) tumors. A recurrent region of 34.5 Mb loss which maps to 16q12.2-qter was detected in 5 tumors (1, 6, 9, 21, and 27P) (Figure 2A and Additional file 2: Figure S1). This region encompasses tumor suppressor genes including *CDH1*, *E2F4*, *CTCF* and *FOXF1*. CN losses on the long arm of chromosome 11 were detected in 23% of the tumors (7/30). These tumors shared a recurrent region of 35.5 Mb at 11q22.1-qter (Table 1; Figure 2B and Additional file 2: Figure S1). This region encompasses several known tumor suppressor genes with a role in apoptosis such as *SDHD* and members of the cysteine-aspartic acid protease (caspase) family including *CASP4* and *CASP12*. Whole chromosome 9 loss was found in two tumors and segmental losses in four tumors with recurrent regions at 9pter-p13.2 and 9p13.1-11.2. Loss of entire chromosome 13 was found in one metastasis and two primary tumors showed deletions at 13q11-ter and 13q13-q21.32.

### Copy number gains

Recurrent CN gains were found on chromosome 4 (27%), 5 (23%), 7 (13%), 14 (30%) and 20 (37%) (Table 2; Figure 2C and Additional file 2: Figure S1). Several genes involved in tumor development including a variety of growth factors are located on these chromosomes. These include for example *FGF2, FGFB, FGFR3* on 4q; *PDGFR, APC*, *TGFB1*, *SMAD5* on 5; *BRAF, IGFBP3, MDR1* on chromosome 7; *AKT1, BCL2L2, MAX, MMP14*, *DAD1* and *DICER1* on 14, and *E2F1*, *CDH4*, *LAMA5*, *TNFRSF6B* on chromosome 20.

A MOR of 230 kb gain at 14q11.2 downstream of the *DAD1* locus was observed in four tumors (20, 23, 27M, 28) which overlapped with partial or entire gains in five other tumors (1, 7, 25, 27P, 32). This region encompassed several genes among others *DHRS4L2*, *REC8L1*, *IPO4*, *PSME1* and *PCK2*. One tumor (case 25) displayed gain of entire chromosome 14, and four tumors (1, 20, 23, 27M) carried partial or small gain of 14 without harboring copy number losses on chromosome 18. A MOR of 1.4 Mb gain at the telomeric region of chromosome 20 (q13.33) was detected in 9/30 cases (30%) (data not shown). The 20q13.33 region overlapped with several cancer related genes such as *CDH4*, *LAMA5*, *RPS21*, *KIAA1510*, *TNFRSF6B* (*M68*, *DcR3*), *RTEL*, *EEF1A2* and *PTK6.*

### Verification of recurrent CNAs by qPCR

All CNAs identified by a-CGH were reviewed to identify recurrent loss or gain regions. The selection of candidate genes for validation were based on the most common CNAs in the present study as well as candidate genes identified from the literature [5,12,14-17,25] and their potential role in tumorigenesis of SI-NETs. To validate CNAs in the selected recurrent regions, qPCR-based copy number analysis was used. For validation of CN losses in chromosome 18, we selected *EMILIN2* (p11.32-31), *DCC* (q21.1-2), *BCL2* (q21.33) and *CDH19* (q22.1). CNs in chromosome 18 were verified in 12/19 (63%) of the tumors for *EMILIN2*, 11/18 (61%) for *DCC*, 11/17 (65%) for *BCL2* and 11/18 (61%) for *CDH19* (Additional file 3: Table S2). Consistent results were obtained when two different assays used for the same gene (*DCC, CDH19*).

For losses on chromosomes 16 and 11, we selected *CDH1* on 16q22.1 and *SDHD* on 11q23.1 and they were verified respectively in 3/5 (60%) and 4/7 (57%) of tumors with corresponding losses detected by a-CGH (Additional file 3: Table S2). Taken together CNAs and CNs detected by a-CGH were confirmed by qPCR in 61% and 78% of tumors respectively (Additional file 3: Table S2).

In addition to the tumor panel screened by aCGH, qPCR-based CN profiling was performed in additional SI-NETs (Table 3). Overall, qPCR was carried out for 11 tumor pairs of which 9 were primary tumors with paired metastases from the same patients and two were paired metastases from the same individual (Table 3). CNAs for the target genes detected by a-CGH and qPCR were similar in primary tumors and corresponding metastases in most patients, however, small differences were observed at some loci.

**Table 3:** Copy numbers detected by a-CGH/q-PCR in paired tumors (Matched primary - metastasis or two metastasis)

**Case no.**	**Sample studied**	***EMILIN2 18p11.32-31***	***DCC 18q21.2***	***BCL2 18q21.33***	***CDH19 18q22.1***	***CDH1 16q22.1***	***SDHD 11q.23.1***
20	P	2/2	2/2	2/2	2/2	2/2	2/2
20	M (reg)	-/2	-/2	-/2	-/2	-/2	-/2
21	M1 (ova)	-/1	-/1	-/1	-/1	-/1	-/1
21	M2 (ova)	1/2	1/2	1/1	1/1	1/2	1/1
23	P	-/2	-/2	-/2	-/2	-/2	-/2
23	M (reg)	2/2	2/2	2/2	2/2	2/2	2/2
24	M (reg)	-/1	-/1	-/1	-/1	-/2	-/2
24	M (reg)	1/1	1/1	1/1	1/1	2/2	2/2
26	P	1/1	1/2	1/2	1/2	2/2	2/2
26	M (reg)	-/2	-/2	-/2	-/2	-/1	-/2
27	P	1/2	2/2	2/2	2/2	1/2	1/2
27	M (reg)	2/2	2/3	2/2	2/3	2/2	2/2
28	P	-/1	-/1	-/1	-/1	-/2	-/2
28	M (reg)	1/1	1/1	1/1	1/2	2/2	2/2
29	P	2/2	2/3	2/2	2/3	2/2	2/2
29	M (reg)	-/2	-/2	-/2	-/2	-/2	-/2
30	P	-/1	-/2	-/1	-/1	-/2	-/2
30	M (reg)	1/1	1/2	1/1	1/2	2/2	2/2
31	P	1/1	2/2	2/2	2/2	2/1	2/1
31	M (reg)	-/1	-/2	-/2	-/2	-/1	-/2
32	P	-/1	-/1	-/1	-/1	-/2	-/2
32	M (reg)	1/1	1/1	1/1	1/1	2/2	2/2

P = primary; M = metastasis; ova = ovarial; reg = regional 1 = loss; 2 = no abnormality; 3 = gain; - = not analyzed.

Chomosomal locations are according to the ensembl database (http://www.ensembl.org).

### Clustering analysis and associations to clinical parameters for recurrent regions

**Figure 3:**
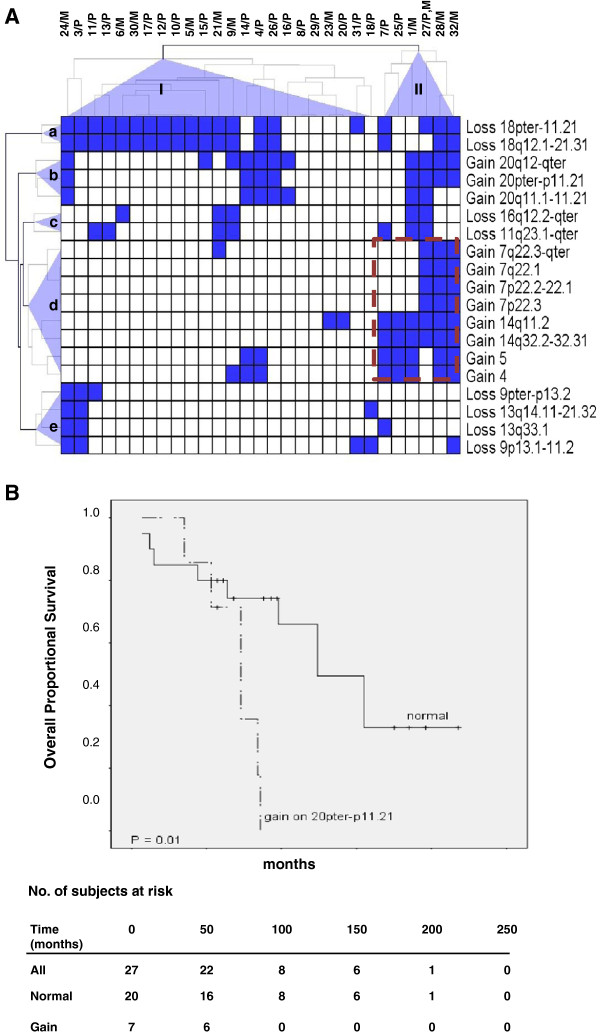
**Unsupervised hierarchical clustering and overall survival for recurrent regions of CNAs in SI-NETs. (A)** Clustering analyses identified two tumor groups (I and II) as well as five chromosomal clusters (a-e). CNAs detected in the primary tumor and metastasis of case 27 were pooled to prevent magnification of alterations in multiple samples from the same case. **(B)** Kaplan-Meier plots showing shorter overall survival for cases with gain in 20pter-p11.21. The table below indicates the number of subjects at risk at different time points during follow-up.

The a-CGH data was subjected to hierarchical cluster analysis to detect tumor groups with similar patterns of chromosomal alterations based on recurrent regions of CN loss or gain. Two different tumor groups (I and II) and five chromosomal clusters -(a-e) were identified (Figure 3). Tumor group I consisted of 23 tumors (7 metastases, 30%) and group II contained 7 tumors (4 metastases, 56%). Losses in chromosomes 18, 16 and 11 and gain of chromosome 20 were found in both tumor groups. Tumor group II was significantly enriched for all alterations which were part of cluster-d including gains of chromosome 4 (*P* = 0.007), 5 (*P* = 0.003), 7p22.3 (*P* = 0.001), 7p22.2-22.1 (*P* = 0.001), 7q22.1 (*P* = 0.001), 7q22.3-qter (*P* = 0.006), 14q11.2 (*P* < 0.0005), and 14q32.2-32.31 (*P* < 0.0005). A significant correlation was also observed between group II and gain on 20pter-p11.21 (*P* = 0.014).

Cases with extra-hepatic metastases (10, 21, 25, 27 and 32; Additional file 1: Table S1), had a higher likelihood to segregate into group II (*P* = 0.016) and harbored gains on 7p22.3 (*P* = 0.018), 7p22.2-22.1 (*P* = 0.018), 7q22.1 (*P* = 0.018), 7q22.3-qter (*P* = 0.003), 14q11.2 (*P* = 0.049), and 14q32.2-32.31 (*P* = 0.016). Loss on 16q12.2-qter was more common in distant metastases compared with primary tumors (*P* = 0.003), and this loss along with gain on 7q22.3-qter were more common in metastases compared to primary tumors (*P* = 0.016 and *P* = 0.047, respectively), suggesting their involvement in tumor progression.

Next, we tested possible associations between CNAs in recurrent regions and patient survival. CN gain in the 20pter-p11.21 region was associated with shorter overall survival (*P* = 0.013) (Figure 3B). Cox proportional hazards modeling supported this finding as no confounders were found. No other significant associations were observed between recurrent CNAs and survival. Losses on 9p and 18p as well as gain on 7q were associated with younger age at diagnosis (*P* = 0.025-0.029). CN losses on chromosomes 11 and 16 and gain on chromosome 20 were more frequent in female patients involving 11q23.1-qter (*P* = 0.039), 16q12.2-qter (*P* = 0.019), 20pter-p11.21 (*P* = 0.017) and 20q11.1-11.21 (*P* = 0.039).

## Discussion

In this study we report frequent CN losses of chromosome 18 as well as of chromosomes 16q, 11q, 13 and 9 and gains of chromosomes 20, 14, 4, 5 and 7. These findings are in line with another Swedish study using a similar platform to identify CNAs in SI-NETs [15], and using a parallel exome sequencing approach to find regions of recurrent losses and gains and mutated candidate genes therein [26]. For some recurrent regions CNAs were more frequently observed in metastases than in primary tumors such as loss of 16q (33% vs. 6%) and gain of 7q (25% vs. 6%) (Table 4). The observations of more genetic aberrations in metastases compared to primary tumors indicate that genetic changes accumulate during tumor progression.

**Table 4:** Comparison of CNAs by a-CGH in recurrent regions in primary tumors and metastases

**Recurrent region**	**Detected in [number of cases / informative (%)]**		
	**Primary tumors**	**Metastases**	**All tumors**
*No of samples*	n = 18	n = 12	n = 30
*Losses*			
9pter-p13.2	2 / 18 (11%)	1 / 12 (8%)	3 / 30 (10%)
9p13.1-11.2	3 / 18 (17%)	2 / 12 (17%)	5 / 30 17%)
11q23.1-qter	4 / 18 (22%)	3 / 12 (25%)	7 / 30 (23%)
13q14.11-21.32	2 / 18 (11%)	1 / 12 (8%)	3 / 30 (10%)
13q33.1	2 / 18 (11%)	1 / 12 (8%)	3 / 30 (10%)
16q12.2-qter	1 / 18 (6%)	4 / 12 (33%)	5 / 30 (17%)
18pter-11.21	12 / 18 (67%)	8 / 12 (67%)	20 / 30 (67%)
18q12.1-21.31	10 / 18 (56%)	8 / 12 (67%)	18 / 30 (60%)
*Gains*			
4pter-qter	4 / 18 (22%)	4 / 12 (%)	8 / 30 (27%)
5pter-qter	4 / 18 (22%)	3 / 12 (25%)	7 / 30 (23%)
7p22.3	1 / 18 (6%)	2 / 12 (17%)	3 / 30 (10%)
7p22.2-22.1	1 / 18 (6%)	2 / 12 (17%)	3 / 30 (10%)
7q22.1	1 / 18 (6%)	2 / 12 (17%)	3 / 30 (10%)
7q22.3-qter	1 / 18 (6%)	3 / 12 (25%)	4 / 30 (13%)
14q11.2	4 / 18 (22%)	5 / 12 (42%)	9 / 30 (30%)
14q32.2-32.31	3 / 18 (17%)	3 / 12 (25%)	6 / 30 (20%)
20pter-p11.21	4 / 18 (22%)	4 / 12 (33%)	8 / 30 (27%)
20q11.1-11.21	5 / 18 (28%)	2 / 12 (17%)	7 / 30 (23%)
20q12-qter	6 / 18 (33%)	5 / 12 (42%)	11 / 30 (37%)

Loss of chromosome 18 was the most prominent alteration observed in 70% of all tumors. This result is in agreement with previous studies and confirms the high frequency of chromosome 18 loss in SI-NETs [12,14,15]. Loss of chromosome 18 was the only detectable recurrent CNA in four tumors of which three were primary, suggesting a potential role in tumor initiation. We identified two recurrent regions of losses of 18p and 18q and a 247 kb MOR of loss at the *EMILIN2* locus within the pter-11.21 interval which was verified by qRT-PCR. *EMILIN2*, which is a component of extracellular matrix, suppresses the growth of cancer cells and has a role in cell survival and apoptosis [27]. In addition, methylation of *EMILIN2* is associated with poor outcome in breast cancer [28]. Another MOR of 2 Mb loss was observed at 18q22.1 in one tumor which overlapped with losses in 17 other tumors. Losses involving18q22.1 have been reported by several groups, and a 40 kb CN variation in 18q22.1 has been reported to be over-represented in SI-NET patients [17]. Furthermore, this region corresponds to one of the three MOR of deletions (R2) described in familial SI-NET [5]. This region encompasses the *CDH19* and *CDH7* genes, however, we could not confirm the loss of *CDH19* in tumor 16 with the small deletion in 18q22.1. This could be due to mixed tumor populations giving variable results for this particular tumor or represent an artifact finding. Mutation analyses of genes in the 18q22.1 region including *CDH7* and *CDH19* did not reveal any tumor specific mutations [5], suggesting that other mechanisms of gene inactivation are operational.

Nine of the 30 tumors (30%) had intact chromosome 18 (Table 1). Three of these (20, 23, 27M) had only recurrent gains of chromosomes 14, two tumors (8, 29) were intact even for other recurrent alterations and the other tumors had gains of 14 together with other alterations such as gains of chromosomes 4 and 5 or losses involving chromosomes 3, 9, 11, 13 and 16. This finding is in agreement with previous reports and supports the suggestion of distinct genetic alterations and pathways in SI-NETs [15,16]. However, a mutually excluding relationship between loss of 18 and gain of 14 observed in another cohort [15], was not revealed in our study. Furthermore we did not find an association between gain of 14 and poor survival, as reported in another cohort [15].

Four of 12 (33%) metastases had loss at 16q as compared to 1/18 (6%) primary tumors confirming our previous observations by conventional CGH [12], and suggesting a role in SI-NET progression. By contrast, another study reported that losses on 16q are more frequent in primary tumors than in metastases [15]. This discrepancy could be due to the different type of metastases used in different studies. A possible role of chromosome 16 loss in tumor progression is supported by studies of other tumor types, e.g. advanced prostate cancer and relapses of Wilm’s tumor showing frequent LOH in 16q [29]. Using qPCR we confirmed CNAs at the *CDH1* gene locus, located in the MOR of 561 kb at 16q22.1, in 60% of cases. *CDH1* is inactivated by mutation or promoter hypermethylation in e.g. gastric and breast cancers [30,31]. Inactivation of *CDH1* is associated with its dysfunction in cell-cell adhesion as well as triggering of cancer invasion and metastasis. We have also observed promoter hypermethylation of *CDH1* in SI-NETs (Fotouhi et al., unpublished data). Thus, *CDH1* could represent a potential candidate tumor suppressor gene in this region.

CN losses within 11q have been reported for many cancer types and have been associated with metastatic disease for example in pheochromocytoma [32]. Loss of 11q is also linked to a high risk of relapse in neuroblastoma and poor clinical outcome in oral cancer [33,34]. In the present study, losses of 11q were frequently observed at almost similar frequencies in primary tumors (22%) and metastasis (25%).

In addition to gain of entire chromosome 20, a 1.4-4.2 Mb region at 20q13.33 was gained in about 33% of tumors. Similar alterations have been reported in e.g. digestive tract tumors and breast cancer [35,36], and have been correlated with lymph node metastasis in gastric cancer [37]. Furthermore, the 20q13 region has been reported as the most commonly amplified region in cancer and 13 of the amplified genes were proposed as “cancer initiating genes” [38]. Several cancer-related genes are located on 20q13. *TNFRSF6B*, that may inhibit apoptosis and promote cell survival, is over-expressed in gastrointestinal tract tumors [39], colorectal carcinoma [40] and gastric cancer [41]. Furthermore cases with gain of 20pter-p11.21 exhibited shorter overall survival, supporting a role of this alteration in aggressive SI-NET. However, since only 7 cases showed gain of this region, the association is based on a limited number of cases and firm conclusions would require analysis of additional cases.

Unsupervised clustering of all CNAs identified two distinct tumor groups (I and II) and 5 chromosomal clusters (a-e). Interestingly, gains of chromosomes 4, 5, 7 and 14 clustered together in tumor group II. This distinct genetic alteration in SI-NETs is in accordance with a previous study [16]. Four metastases including two distant metastases (21, 27) which harbored losses of 16q, clustered together with loss of chromosome 11 (cluster-c). We investigated if different tumor groups and chromosomal clusters are associated with clinical variables or tumor types. As shown in Figure 3, clusters-d and b alterations (gains of 4, 5, 14 and 20) in tumor group II comprised a higher number of metastases (57%) than group I (32%). This finding suggests a possible role for gain of chromosomes 4, 5, 14 and 20 in progression of primary tumors to metastases in SI-NETs. In addition tumors with gain of 20pter-p11.21 were also associated with tumor group II (*P* = 0.014) and the patients had a shorter overall survival (*P* = 0.013). Female patients more frequently showed losses on chromosomes 11q and 16q and gains on 20p and 20q. Furthermore, gain on 7q and loss on chromosomes 9p and 18p were associated with younger age at diagnosis.

## Conclusions

We identified two recurrent regions on chromosome 18 with a possible role in tumor initiation. Recurrent CN gains of chromosomes 4, 5, 7, 14 are usually concomitant and could together with loss of 16q and gain of 20p be involved in progression of SI-NETs. Distinct genetic alterations and pathways are involved in SI-NET development.

## Abbreviations

SI-NET: Small intestinal neuroendocrine tumor; a-CGH: Array comparative genomic hybridization.

## Competing interests

The authors declare that they have no competing interest.

## Authors’ contributions

JH contributed to the study design, performed a-CGH experiments and data analysis, interpreted the results and drafted the manuscript. OF performed qPCR, clustering, statistical analysis and interpreted the results. LS performed a-CGH experiments. MK, AH, CL and JZ provided the clinical information. OF, LS, AH and JZ critically revised the manuscript. CL contributed to conception and study design, interpretation of data and substantially revised the manuscript. All authors read and approved the final manuscript.

## Pre-publication history

The pre-publication history for this paper can be accessed here:

http://www.biomedcentral.com/1471-2407/13/505/prepub

## Supplementary Material

Additional file 1: Table S1 Clinical data for the 32 patients with SI-NET in the study. Click here for file

Additional file 2: Figure S1 Examples of a-CGH profiles showing loss of 16q12.1-ter in case 1, loss of 11q22.1-qter in case 27P, gain of 14q11.1-32.31 in case 32 and gains of 14q11.2 and q32.2-qter in case 28. Click here for file

Additional file 3: Table S2 Copy numbers detected by a-CGH and q-PCR (aCGH/q-PCR). Click here for file
