# Supplementary material for: Copy number alterations in small intestinal neuroendocrine tumors determined by array comparative genomic hybridization
Source: BMC Cancer. 2013 Oct 29;13:505. doi: 10.1186/1471-2407-13-505 (PMC3819709; doi:10.1186/1471-2407-13-505)
Supplement: Additional file 2: Figure S1 — Examples of a-CGH profiles showing loss of 16q12.1-ter in case 1, loss of 11q22.1-qter in case 27P, gain of 14q11.1-32.31 in case 32 and gains of 14q11.2 and q32.2-qter in case 28. [file 1471-2407-13-505-S2.ppt]

## Slide 1
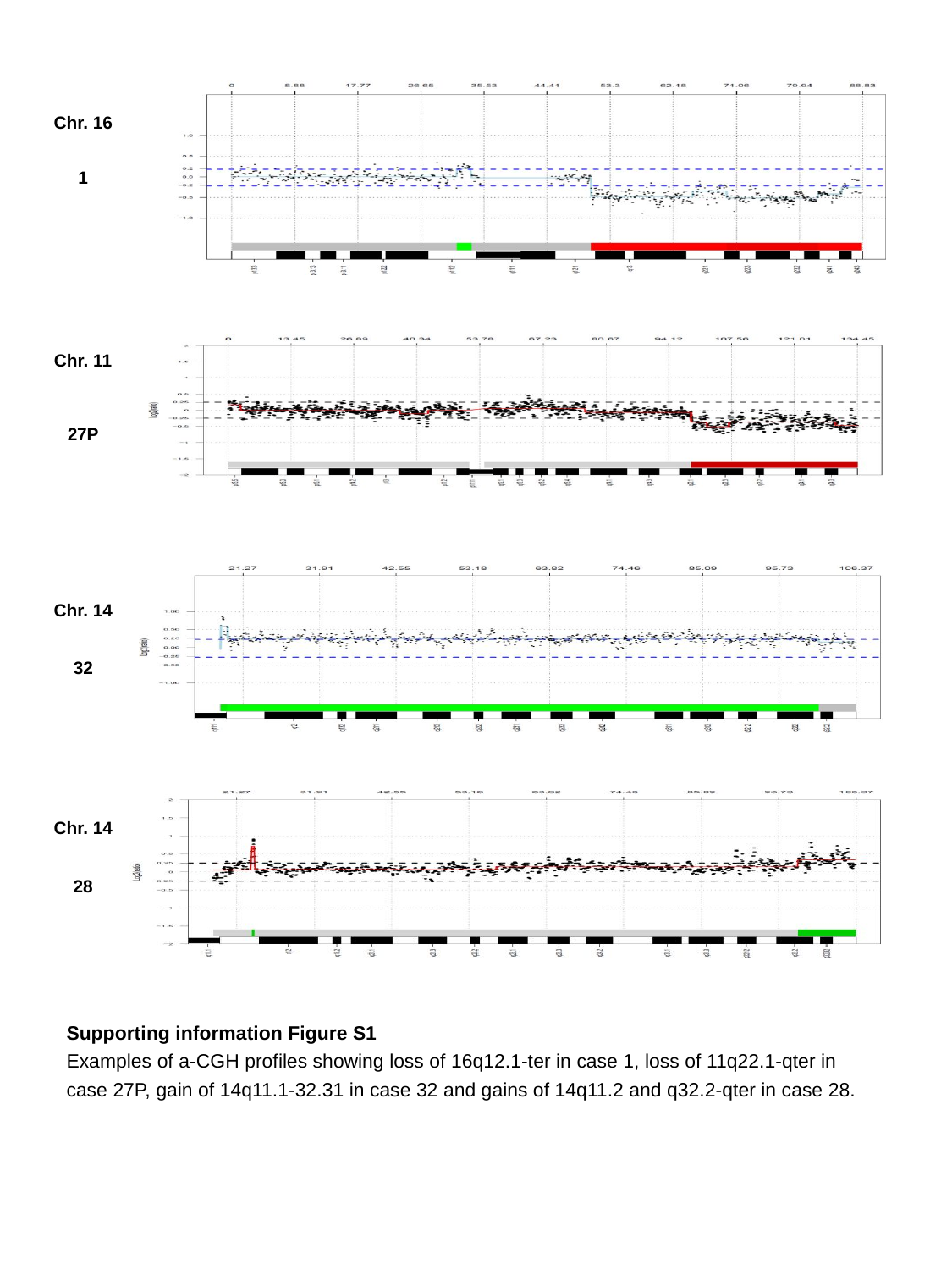

Chr. 16
1
Chr. 11
27P
Chr. 14
32
Chr. 14
28
Supporting information Figure S1
Examples of a-CGH profiles showing loss of 16q12.1-ter in case 1, loss of 11q22.1-qter in case 27P, gain of 14q11.1-32.31 in case 32 and gains of 14q11.2 and q32.2-qter in case 28.
